# Supplementary figures and images for: RIPK1 is required for ZBP1-driven necroptosis in human cells
Source: PLoS Biol. 2025 Feb 21;23(2):e3002845. doi: 10.1371/journal.pbio.3002845 (PMC11844899; doi:10.1371/journal.pbio.3002845)

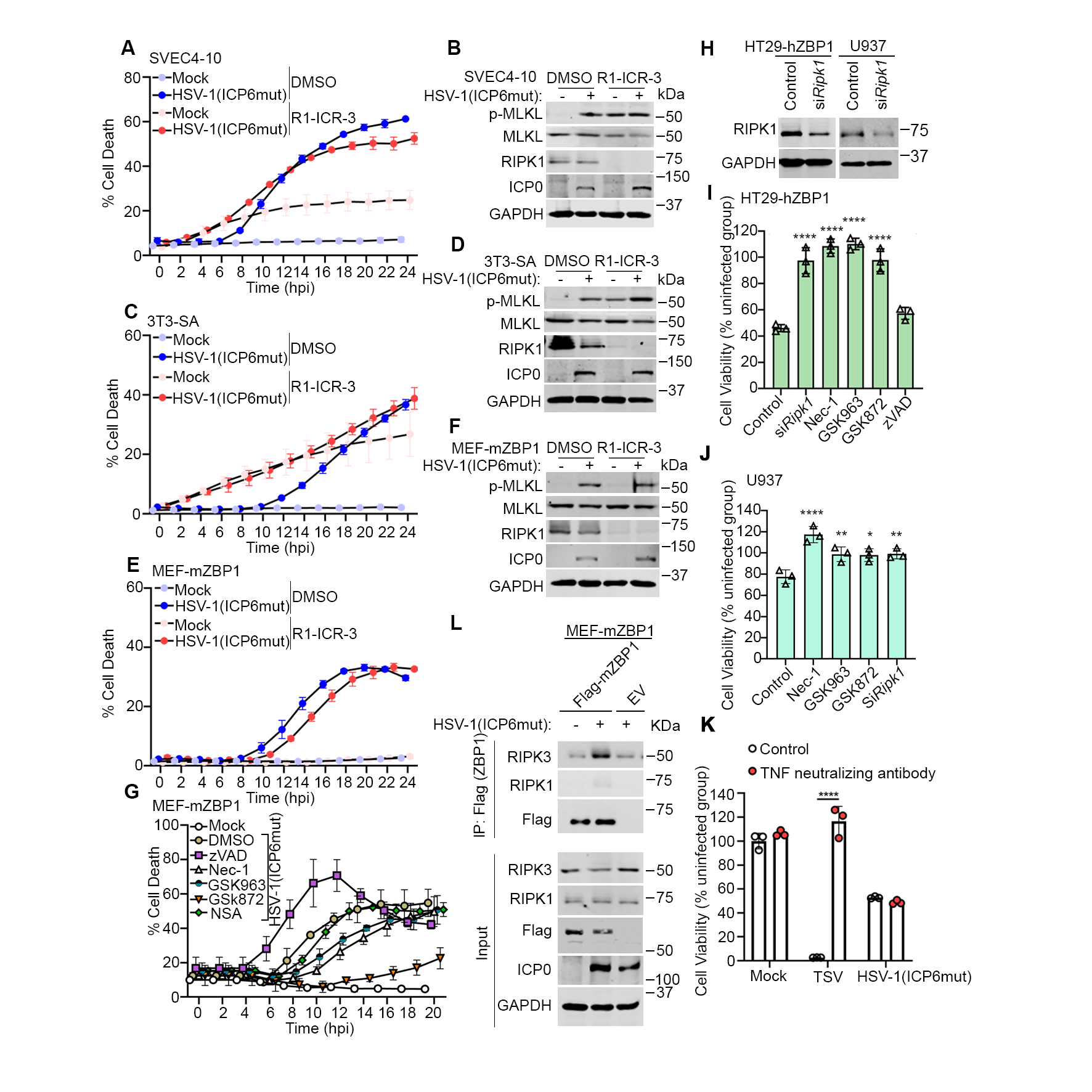

Supplement: S1 Fig — (A–F) Cell death kinetics of SVEC4-10 (A), 3T3-SA (C), and MEF-mZBP1 (E) cells treated with DMSO or R1-ICR-3 (1 μm) for 5 h, followed by HSV-1(ICP6mut) infection. Immunoblotting of SVEC4-10 (B), 3T3-SA (D), and MEF-mZBP1 (F) cells pretreated with DMSO or R1-ICR-3 (1 μm) for 5 h, followed by HSV-1(ICP6mut) infection and subjected to immunoblotting with p-MLKL, MLKL, RIPK1, ICP0, and GAPDH antibodies. (G) Cell death kinetics of MEF-mZBP1, followed by HSV-1(ICP6mut) infection, with or without Nec-1, GSK963, GSK872, NSA, or zVAD. (H) Validation of siRIPK1 efficiency of down-regulation of RIPK1 in HT29-hZBP1 and U937 cells. (I) HT29-hZBP1 cells were transfected with either scramble (control) or RIPK1 siRNA for 48 h, followed by HSV1mutRHIM infection. The RIPK1 siRNA-treated group was left with DMSO treatment. Control siRNA-transfected groups were treated with Nec-1, GSK963, GSK872, or zVAD inhibitors accordingly, with the DMSO-treated group as the control group. Viability was determined by measuring intracellular ATP levels with a CellTiter-Glo luminescent cell viability assay kit (Promega) at 18 hpi. (J) Cell viability of U937 cells transfected with either scramble siRNA or RIPK1 siRNA for 48 h, followed by HSV1mutRHIM infection (MOI = 20), with or without Nec-1, GSK963, or GSK872. Viability was determined at 18 hpi as described in (B). (K) Cell viability of HT29-hZBP1, followed by HSV-1(ICP6mut) infection or TNF+BV6+zVAD (TSV) treatment, with or without TNF neutralizing antibody. (L) MEF reconstituted with empty vector (EV) or Flag-mZBP1, were either mocked or infected with HSV-1(ICP6mut) for 10 h. Co-immunoprecipitation was performed in these cells. *P < 0.1, **P < 0.0001, ***P < 0.001, ****P < 0.0001. Individual data points indicate 3 technical replicates. Results are representative of at least 2 independent experiments. Error bars represent mean ± SD. The underlying data can be found in S2 Data. (TIF) [file pbio.3002845.s001.tif]

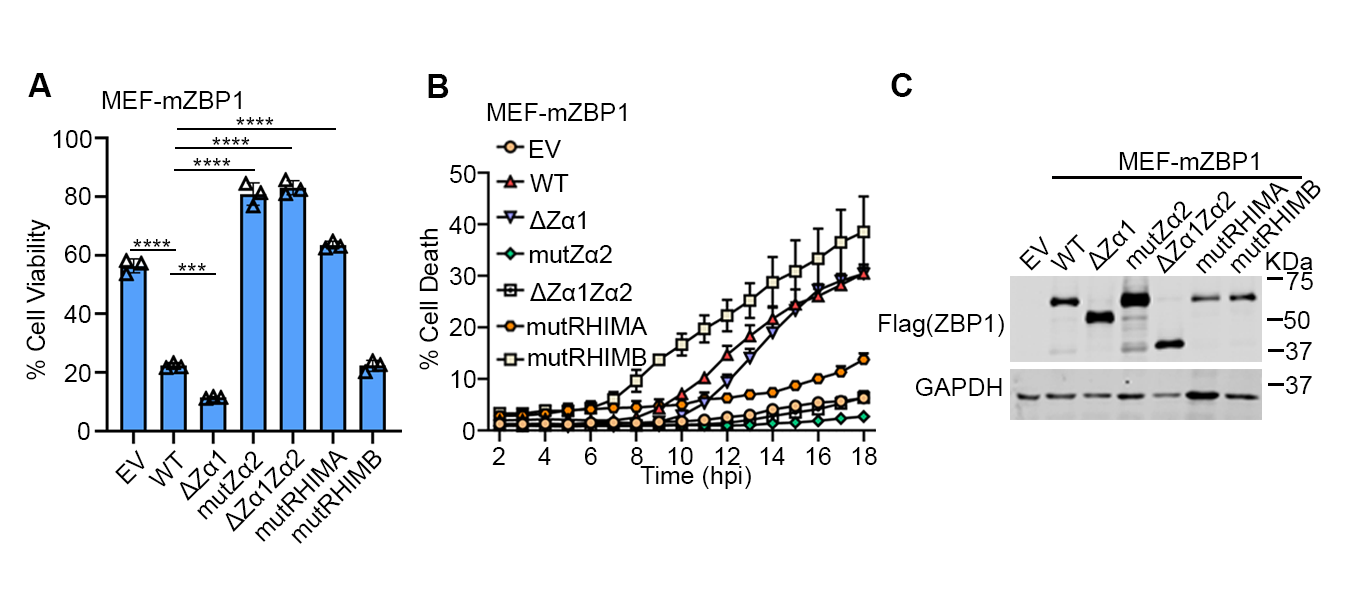

Supplement: S2 Fig — (A) Cell viability of MEFs reconstituted with the indicated mouse ZBP1 constructs, followed by HSV-1(ICP6mut) infection. Viability was determined at 18 hpi by CellTiter-Glo assay. (B) Cell death kinetics of MEFs reconstituted with mZBP1 mutants, followed by HSV-1(ICP6mut) infection. (C) The expression levels of mZBP1 in MEFs reconstituted with mZBP1 constructs were confirmed by immunoblotting with Flag and GAPDH antibodies. One-way ANOVA and Dunnett’s multiple comparisons tests were used to test for statistical differences in (A). *P < 0.1, **P < 0.0001, ***P < 0.001, ****P < 0.0001. Individual data points indicate 3 technical replicates. Results are representative of at least 2 independent experiments. Error bars represent mean ± SD. The underlying data can be found in S2 Data. (TIF) [file pbio.3002845.s002.tif]
