# Supplementary material for: RIPK1 is required for ZBP1-driven necroptosis in human cells
Source: PLoS Biol. 2025 Feb 21;23(2):e3002845. doi: 10.1371/journal.pbio.3002845 (PMC11844899; doi:10.1371/journal.pbio.3002845)

Figure.1D

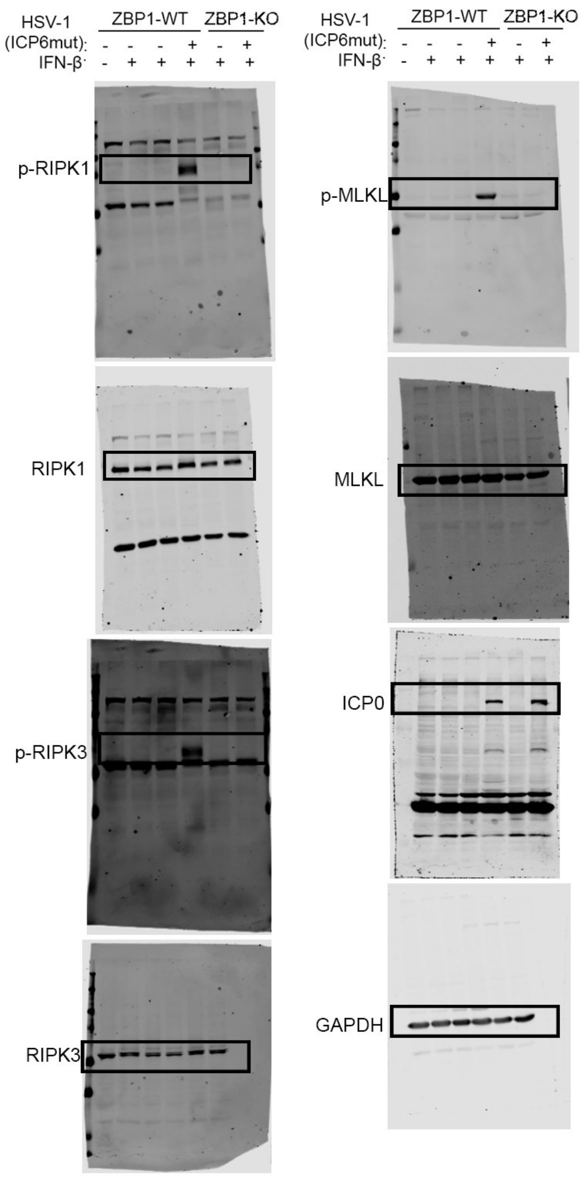

Figure.1B

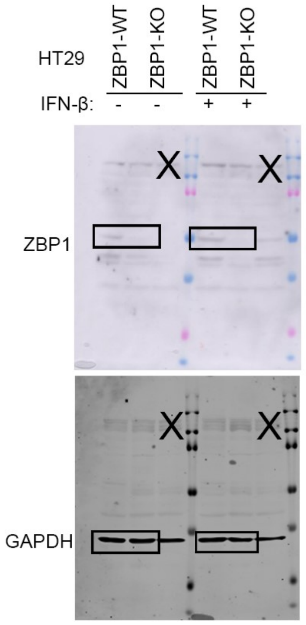

Figure.1F

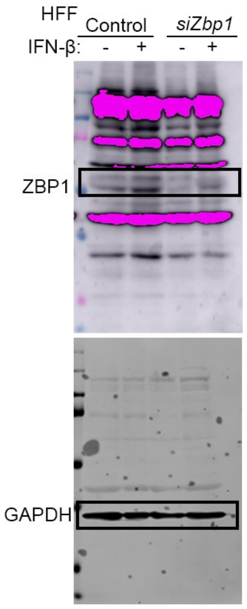

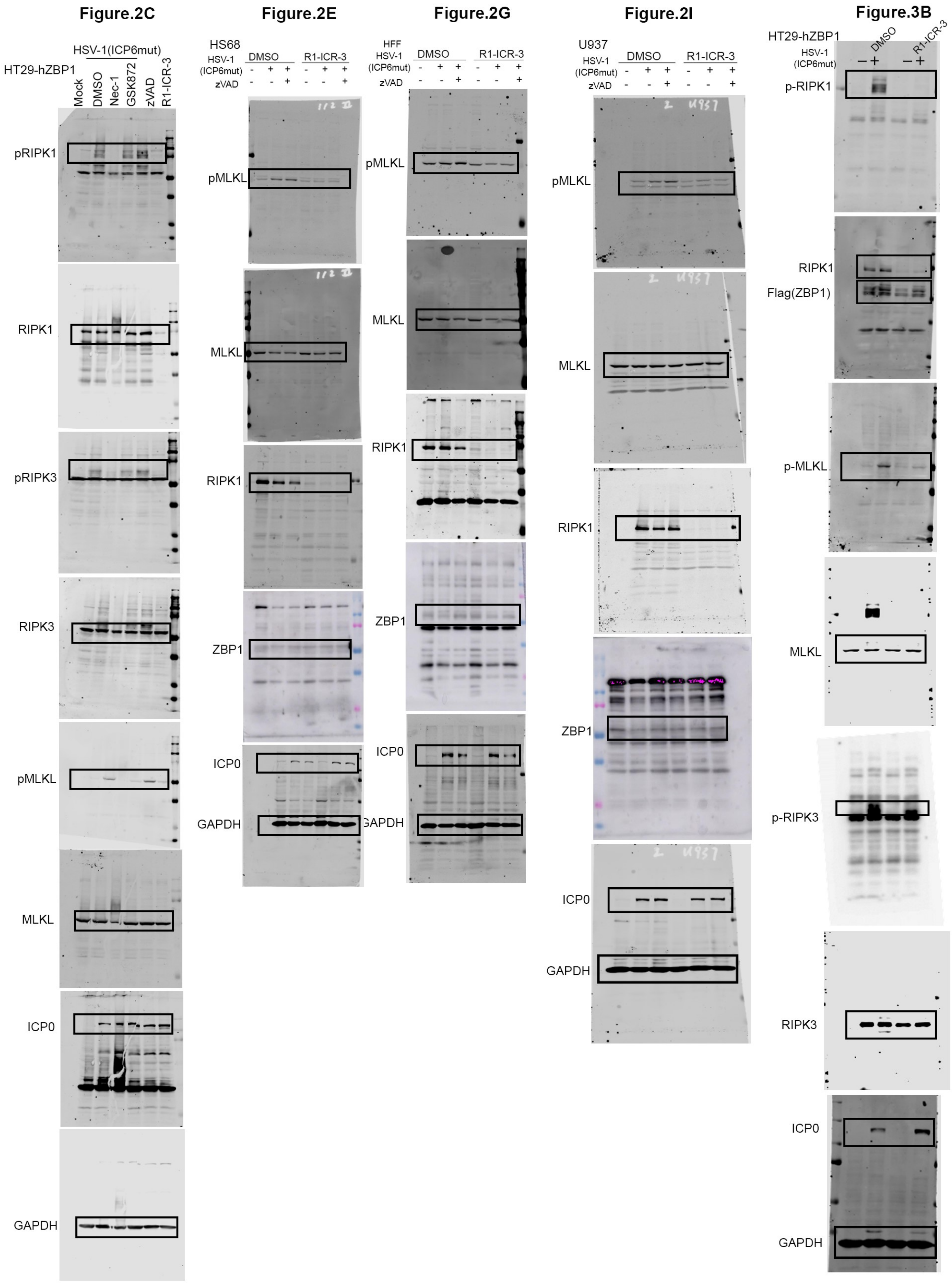

Figure.2C
HT29-hZBP1
HSV-1(ICP6mut)
Mock
DMSO
Nec-1
GSK872
zVAD
R1-ICR-3
pRIPK1
RIPK1
pRIPK3
RIPK3
pMLKL
MLKL
ICP0
GAPDH
Figure.2E
HS68
HSV-1 (ICP6mut)
zVAD
DMSO
R1-ICR-3
pMLKL
MLKL
RIPK1
ZBP1
ICP0
GAPDH
Figure.2G
HFF
HSV-1 (ICP6mut)
zVAD
DMSO
R1-ICR-3
pMLKL
MLKL
RIPK1
ZBP1
ICP0
GAPDH
Figure.2I
U937
HSV-1 (ICP6mut)
zVAD
DMSO
R1-ICR-3
pMLKL
MLKL
RIPK1
ZBP1
ICP0
GAPDH
Figure.3B
HT29-hZBP1
HSV-1 (ICP6mut)
DMSO
R1-ICR-3
p-RIPK1
RIPK1
Flag(ZBP1)
p-MLKL
MLKL
p-RIPK3
RIPK3
ICP0
GAPDH

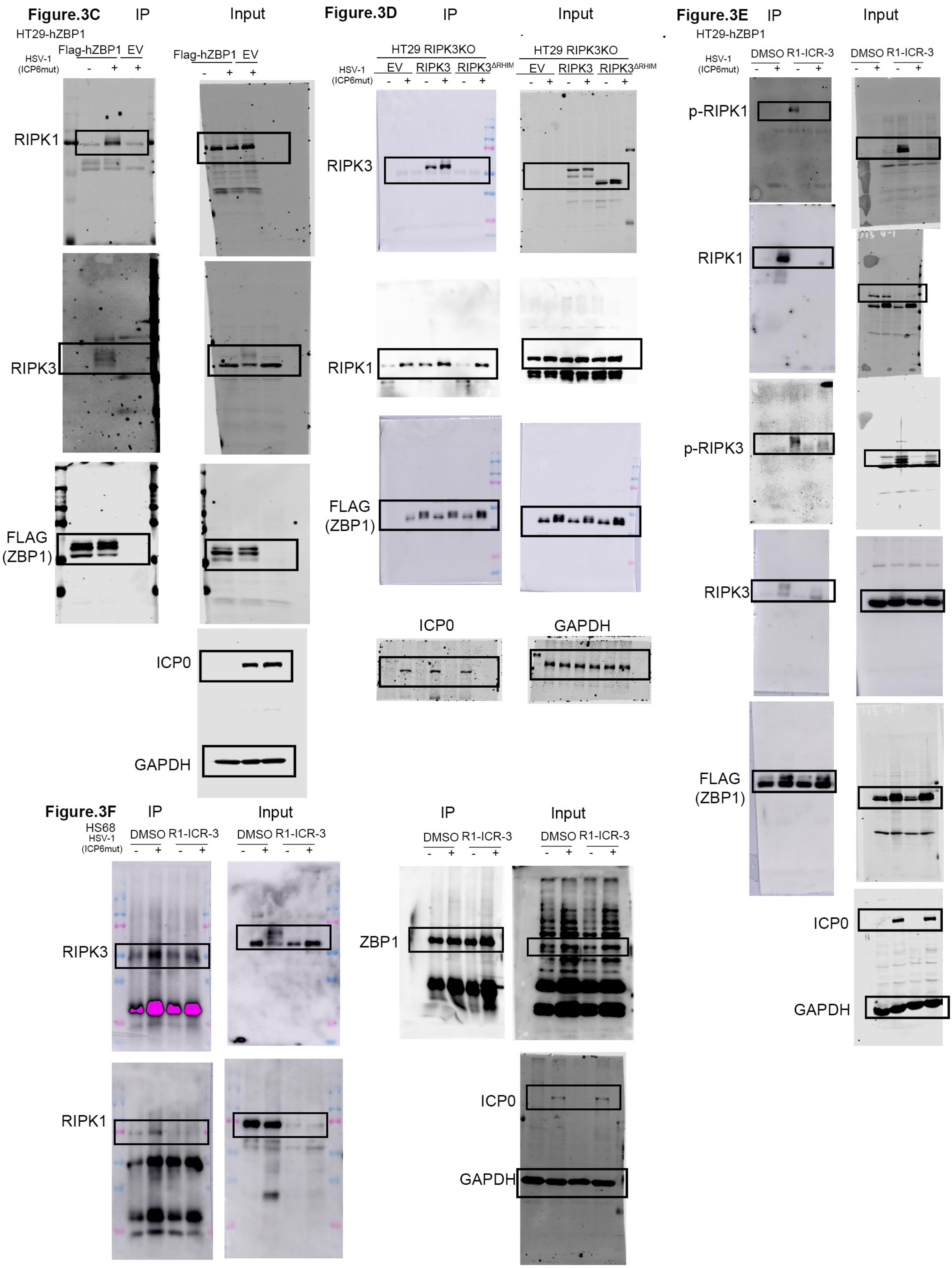

Figure.3C
IP
Input
HT29-hZBP1
HSV-1 (ICP6mut)
Flag-hZBP1 EV
- + +
RIPK1
RIPK3
FLAG (ZBP1)
ICP0
GAPDH
Figure.3D
IP
Input
HT29 RIPK3KO
HSV-1 (ICP6mut)
EV RIPK3 RIPK3ΔRHIM
- + - + - +
RIPK3
RIPK1
FLAG (ZBP1)
ICP0
GAPDH
Figure.3E
IP
Input
HT29-hZBP1
HSV-1 (ICP6mut)
DMSO R1-ICR-3
- + - +
p-RIPK1
RIPK1
p-RIPK3
RIPK3
FLAG (ZBP1)
ICP0
GAPDH
Figure.3F
IP
Input
HS68
HSV-1 (ICP6mut)
DMSO R1-ICR-3
- + - +
RIPK3
RIPK1
ZBP1
ICP0
GAPDH

Figure.4B

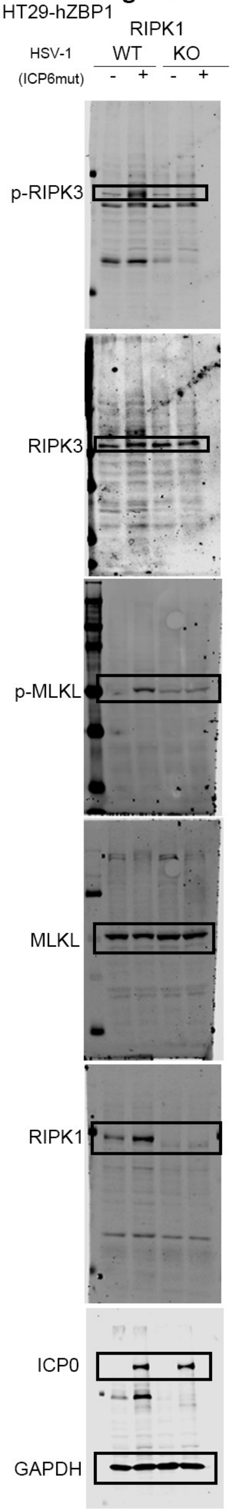

Figure.4G

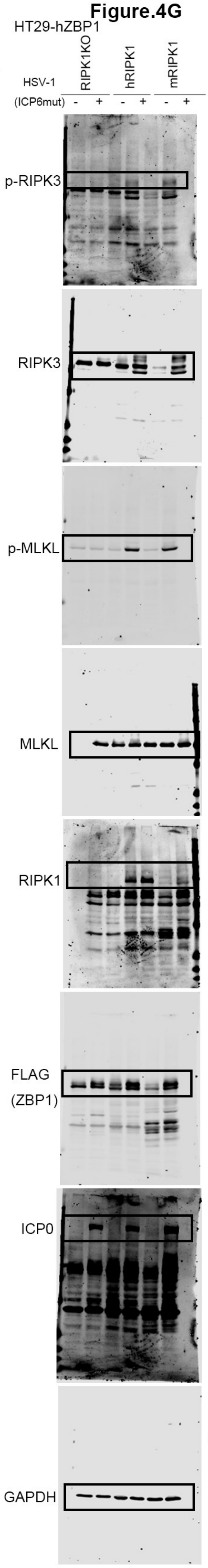

Figure.5C

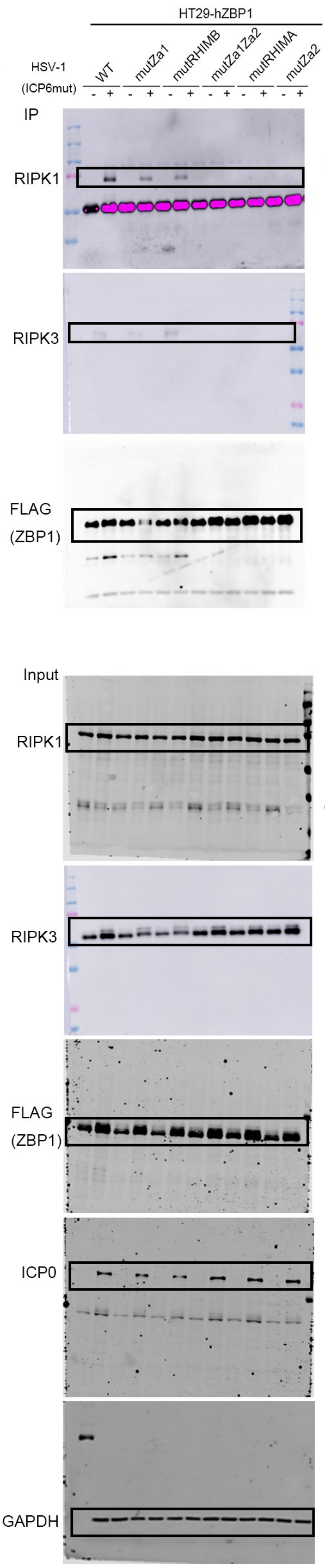

Figure.5F

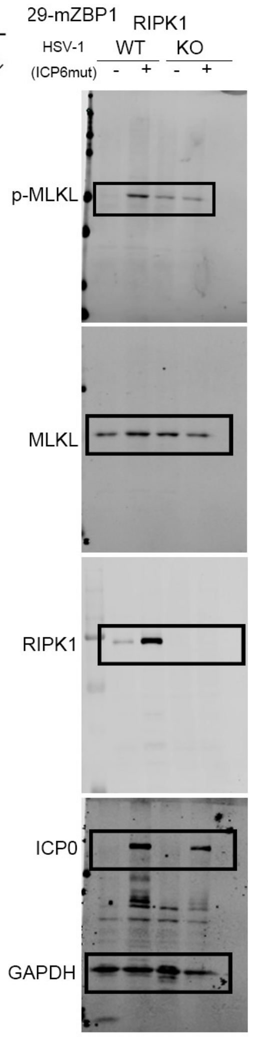

Figure.5G

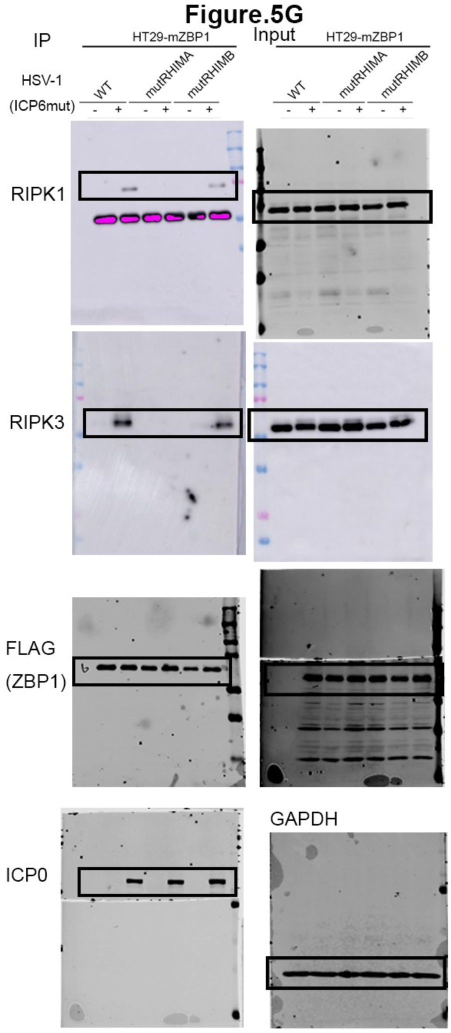

Figure.5I

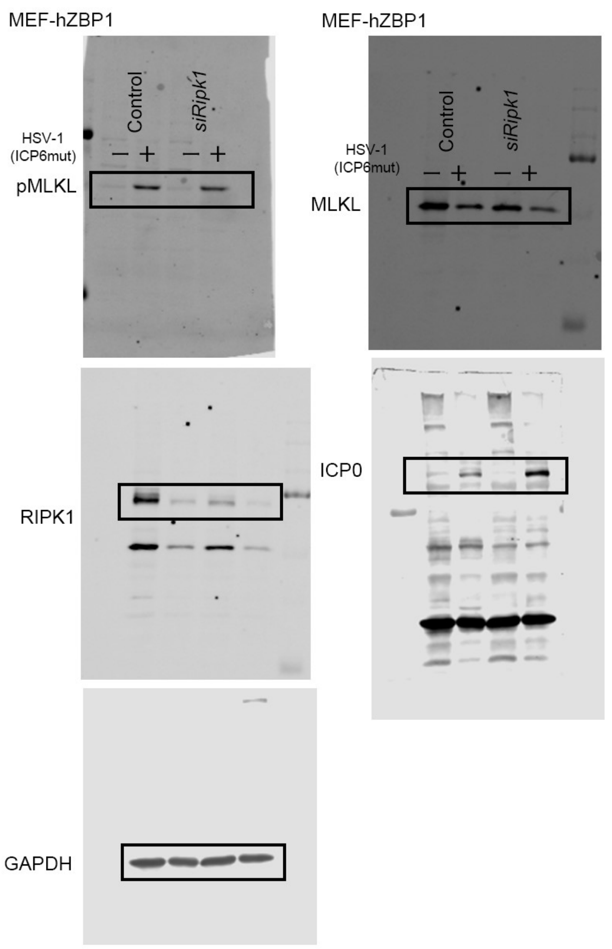

Figure.6G

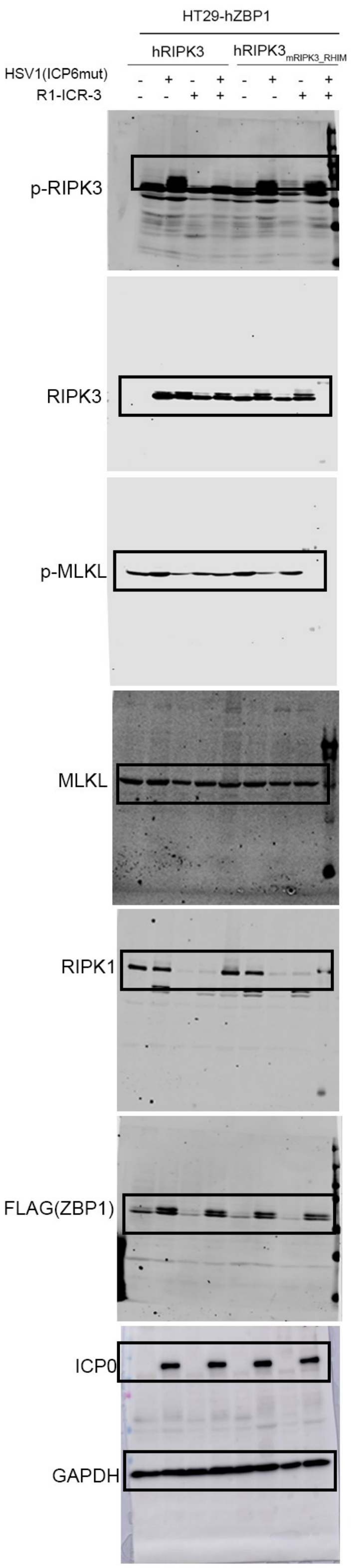

Figure.7B

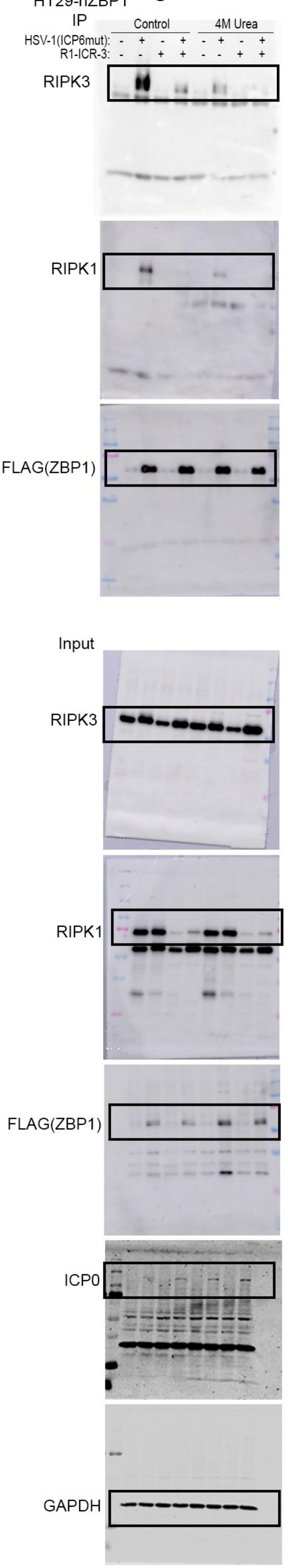

Figure.7C

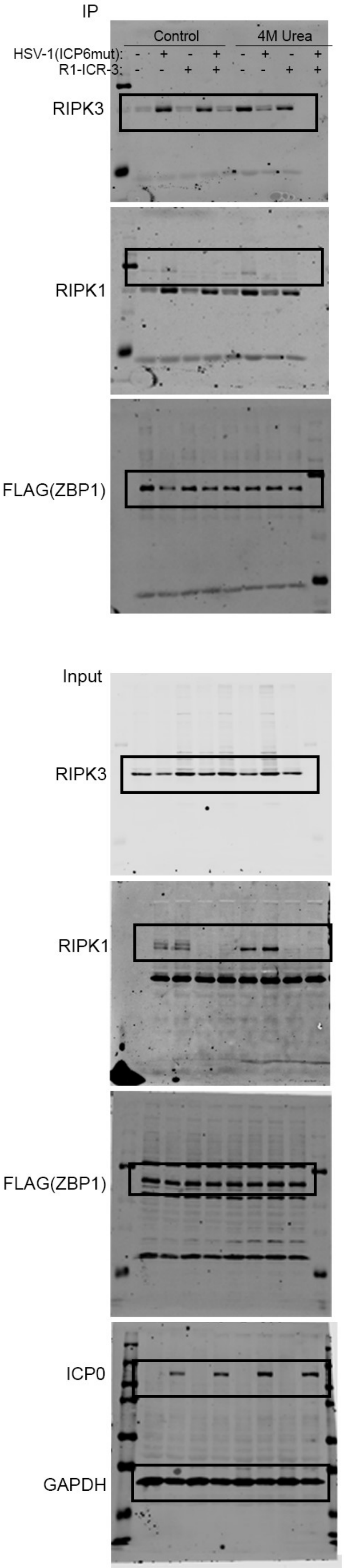

Figure.7D

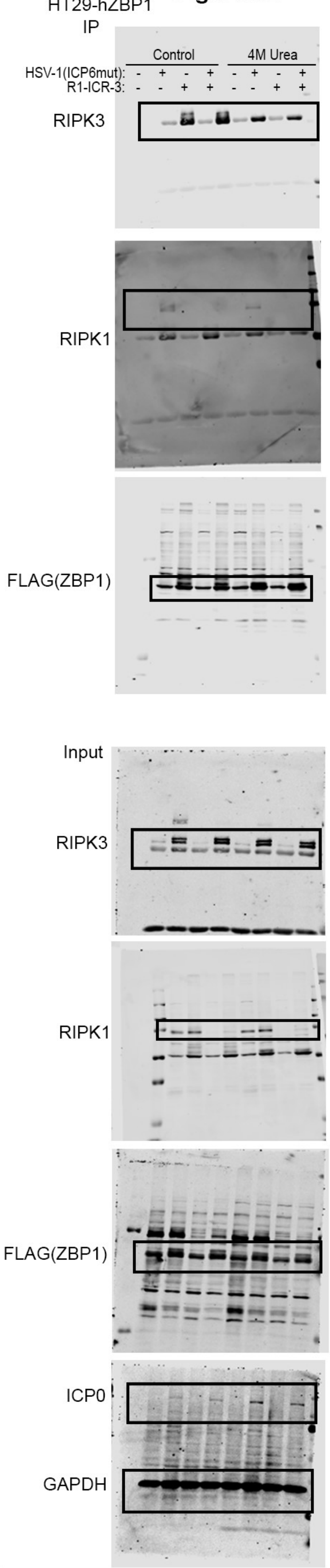

Figure.S1_B

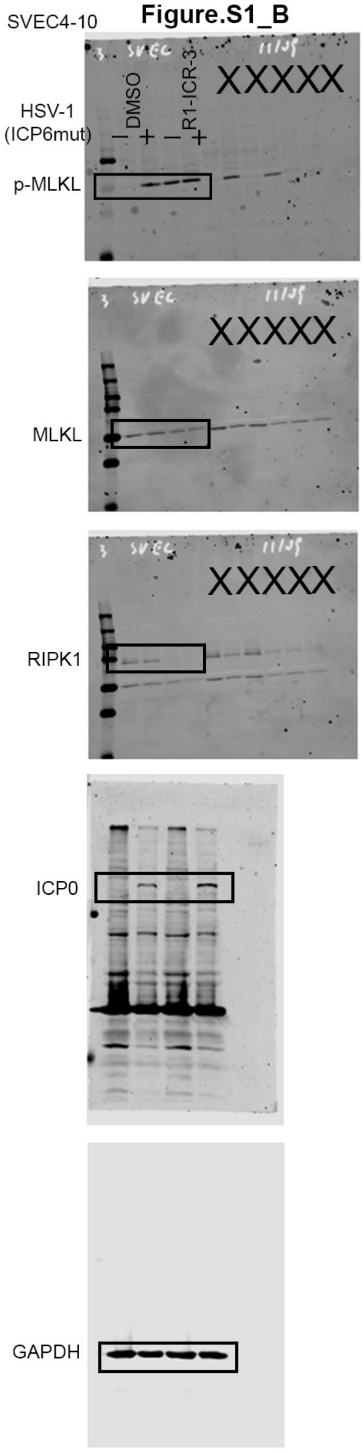

Figure.S1_D

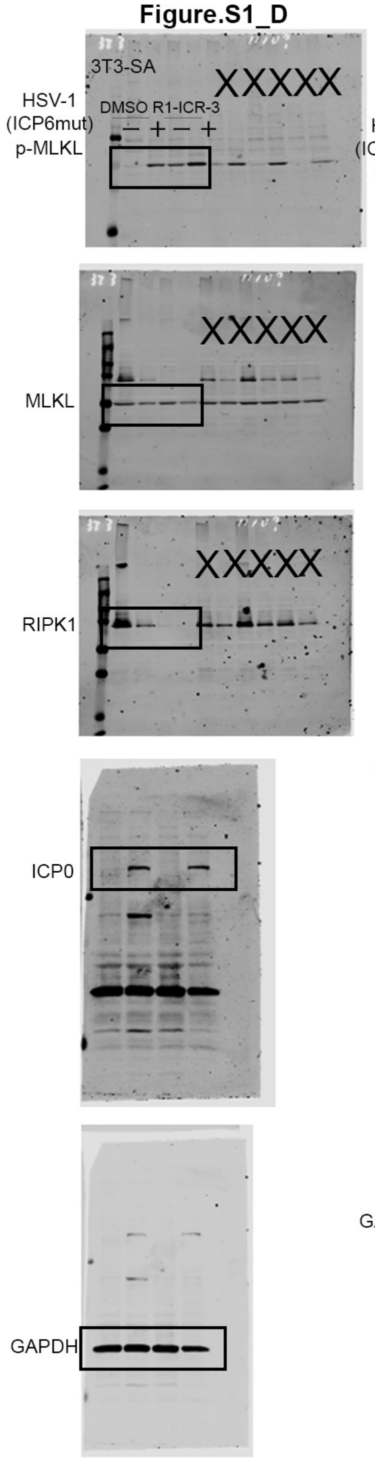

Figure.S1_F

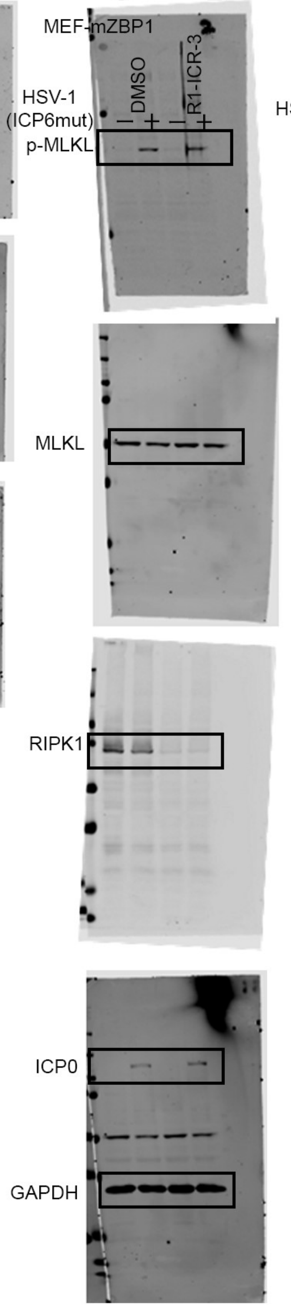

Figure.S1_L

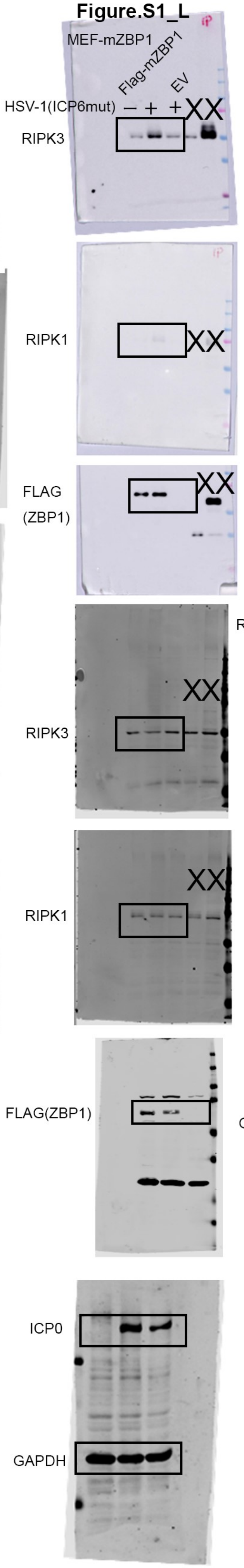

Figure.S1_H

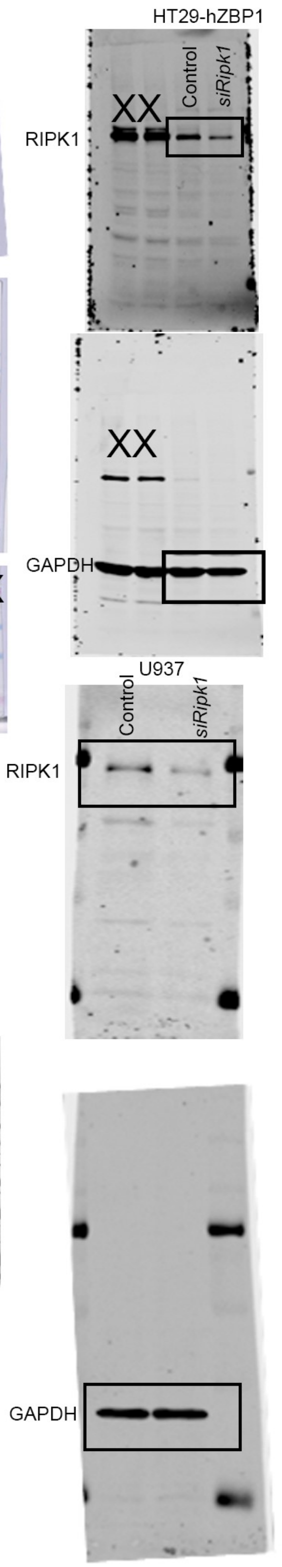

Figure.S2_C

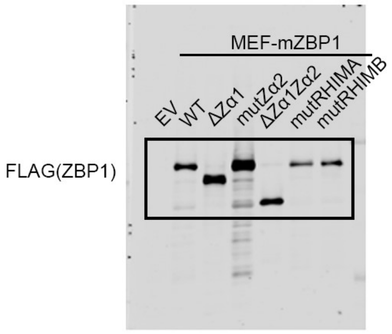

Figure.S2_C

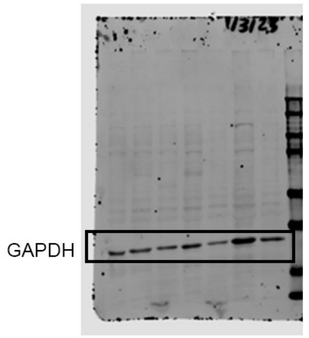

Supplement: S1 Raw Images — (PDF) [file pbio.3002845.s005.pdf]
